# Phenolic-Rich Extracts from Artichoke By-Products Promote Apoptosis in Human Colorectal Cancer Cell Lines

**DOI:** 10.3390/nu18132077

**Published:** 2026-06-25

**Authors:** Rosa Calvello, Antonia Cianciulli, Antonella Compierchio, Chiara Porro, Giusy Rita Caponio, Maria De Angelis, Maria Antonietta Panaro

**Affiliations:** 1 Department of Biosciences, Biotechnologies and Environment, University of Bari Aldo Moro, 70125 Bari, Italy; rosa.calvello@uniba.it (R.C.); a.compierchio2@phd.uniba.it (A.C.); giusy.caponio@uniba.it (G.R.C.); mariaantonietta.panaro@uniba.it (M.A.P.); 2 Department of Clinical and Experimental Medicine, University of Foggia, 71122 Foggia, Italy; chiara.porro@unifg.it; 3 Department of Soil, Plant and Food Sciences, University of Bari Aldo Moro, 70125 Bari, Italy; maria.deangelis@uniba.it

**Keywords:** artichoke by-products, apoptosis, colon cancer, polyphenols, caspase, sustainable bioactive compounds

## Abstract

**Background:** Apoptosis is a fundamental process for maintaining tissue homeostasis, and its dysregulation is closely linked to the development of numerous diseases, including colorectal cancer. In recent years, dietary polyphenols have gained interest due to their antioxidant, pro-apoptotic, and chemopreventive properties. Artichoke (*Cynara scolymus* L.) by-products are rich source of hydroxycinnamic acids and flavonoids, making them promising source of bioactive compounds. **Methods:** In this study we evaluated the cytotoxic and pro-apoptotic activity of four aqueous extracts obtained from artichoke bract by-products, including one commercial hybrid (CAPB) and three local Apulian varieties (BriB, VaMB, LMTB), in human colorectal adenocarcinoma cell lines (Caco-2 and HT29). The extracts were characterized according to their total polyphenol content and phenolic profile. **Results:** The selected artichoke by-product extracts exhibited significant cytotoxic effects both in a concentration- and time-dependent manner, with concentrations ≥ 2 mg/mL significantly reducing cell viability and nearly abolishing it at 4 mg/mL after 48 h. Moreover, treatment with the extracts modulated the expression of apoptosis-related proteins, characterized by an increase in pro-apoptotic markers (Bax, caspase-9, caspase-3) and a decrease in the anti-apoptotic protein Bcl-2, suggesting activation of the mitochondrial apoptotic pathway. In particular, the BriB extract was able to induce an apoptosis rate higher than 80% in Caco-2 cells and achieved comparable rates in HT29 cells at concentrations of 2–3 mg/mL. **Conclusions:** Overall, these findings demonstrate that artichoke by-product extracts exert significant pro-apoptotic effects in colorectal cancer cells and highlight their potential as sustainable sources of bioactive compounds for nutraceutical or adjuvant anticancer applications.

## 1. Introduction

Apoptosis, also called programmed cell death, is an essential biological process in human health and disease. It is a highly regulated protective mechanism that ensures tissue homeostasis in multicellular organisms by eliminating aged, damaged, and/or potentially dangerous cells. This prevents their excessive accumulation in tissues and maintains a physiological balance between cell proliferation and death. Apoptosis also plays a key role in maintaining the constant number of functioning cells and a healthy cellular environment within tissues and organs of the human organism [1,2,3]. However, several studies have reported that dysregulation of the apoptotic process is associated with the onset and development of numerous diseases, including colorectal cancer (CRC) [4,5,6]. Despite advances in screening and therapeutic strategies, CRC remains a leading cause of cancer-related mortality worldwide [6], highlighting the need for innovative preventive approaches targeting key molecular pathways involved in tumor progression. Diet is one of the most important modifiable risk factors for developing CRC. Epidemiological evidence consistently links high consumption of fat and animal protein to an increased risk of cancer, whereas diets rich in fruit and vegetables have protective effects [7,8,9]. This benefit is largely attributed to polyphenolic compounds, which have antioxidant, anti-inflammatory, antiproliferative and pro-apoptotic properties [10,11,12,13]. Artichoke (*Cynara cardunculus* L. var. *scolymus* (L.) Fiori), a polyphenol-rich vegetable widely cultivated in the Mediterranean basin and particularly in Southern Italy, is a valuable source of bioactive compounds. Artichoke bracts contain high levels of hydroxycinnamic acids, particularly derivatives of caffeoylquinic acid such as chlorogenic acid and cynarin, as well as flavonoids including luteolin and apigenin derivatives [14,15]. These phytochemicals are associated with antioxidant, anti-inflammatory, hepatoprotective and lipid-lowering effects [16,17,18,19]. In a recent study, we conducted a comprehensive phenolic characterization of aqueous extracts derived from seven cultivars of artichoke bract by-products, comprising two commercial hybrids and five local varieties cultivated in Apulia, Southern Italy [20]. The results revealed significant differences in phenolic composition depending on the cultivar, highlighting high levels of hydroxycinnamic acids and flavonoids, as well as notable antioxidant and prebiotic properties. Specifically, the selected four varieties exhibited superior free radical scavenging potential and a greater capacity to promote the growth of beneficial bacteria compared to hybrids. These findings demonstrate the nutraceutical value of artichoke agro-industrial by-products and suggest a functional link between their phenolic richness, antioxidant properties and potential benefits for intestinal health. However, although antioxidant and prebiotic activities are relevant in the context of colorectal health, the direct impact of hybrid and local artichoke by-product varieties on CRC cell survival and apoptotic signaling remains poorly investigated.

Therefore, this study builds on previous work by shifting the focus from compositional and functional characterization to targeted biological evaluation in a tumor model. The most promising extracts (one derived from a hybrid and three from local Apulian varieties) were selected based on their phenolic profile, antioxidant activity and prebiotic potential, and their cytotoxic and pro-apoptotic effects were studied in human colorectal adenocarcinoma cell lines (Caco-2 and HT29). Cell viability was assessed, and the expression of key regulators of the intrinsic apoptotic pathway, including Bax, Bcl-2, caspase-9 and caspase-3, was analyzed. This study provides new evidence linking the phenolic composition of artichoke by-product extracts to their ability to modulate mitochondrial apoptotic signaling in CRC cells.

## 2. Materials and Methods

### 2.1. Sample and Extract Preparation

Globe artichoke by-products used in this study were obtained from four genotypes grown under open-field conditions in a common experimental trial established in Ordona (Apulia, Southern Italy; 41°17′58″ N, 15°37′52″ E, 127 m a.s.l.) during the 2022–2023 growing season [20]. The investigated genotypes included one commercial hybrid cultivar, *Capriccio* (CAPB; Nunhems, BASF, Ludwigshafen, Germany), and three local Apulian cultivars, namely *Brindisino* (BriB), *Violetto di Manfredonia* (VaMB), and *Locale di Mola tardivo* (LMTB). Plantlets of the hybrid cultivar were obtained from seed, whereas local cultivars were propagated through offshoots and transplanted in August 2022. Artichoke heads were harvested at commercial maturity during the 2023 production season. After harvest, by-products generated from the processing of the heads, consisting mainly of outer bracts and other non-edible tissues, were collected directly by the authors from the experimental material. Samples were visually inspected to remove damaged, diseased, or mechanically injured tissues and were processed under identical conditions for all genotypes. The selected by-products were transported to the laboratory immediately after collection, stored under refrigerated conditions (4 °C), and processed within a short time to minimize the degradation of bioactive compounds. Samples were prepared and extracted as previously described [20]. Briefly, samples were subjected to aqueous extraction using distilled water as the extraction medium. Briefly, samples were extracted at a biomass-to-solvent ratio of 1:10 (*w*/*v*) under controlled conditions at room temperature. The extraction was carried out under pressures ranging from 5 to 9 bar, applying 10 extraction cycles consisting of alternating static (2 min) and dynamic (2 min) phases, with a 10 s piston stop between cycles, and five percolation steps. The resulting extracts were collected, stored at 4 °C, and subjected to total phenol content (TPC) determination.

### 2.2. Total Phenol Content (TPC) Determination

The TPC of the aqueous extracts was determined using the Folin–Ciocalteu colorimetric assay, following a slightly adapted procedure based on previously described conditions. In brief, 20 μL of the extract, appropriately diluted, was mixed with 980 μL of Milli-Q water. Then, 100 μL of Folin–Ciocalteu reagent was added to the mixture. After an initial reaction time of three minutes, 800 µL of 7.5% *w*/*v* sodium carbonate solution was added to the mixture. The reaction was then allowed to proceed for 60 min in the dark at room temperature. Absorbance was then measured at 720 nm using an Evolution 60s UV-Vis spectrophotometer (Thermo Fisher Scientific, Rodano, Italy). Quantification was performed using a gallic acid calibration curve and the results were expressed as milligrams of gallic acid equivalents per liter of extract (mg GAE/L).

### 2.3. Identification of Phenolic Compounds by UHPLC-MS/MS

Identification of phenolic compounds were performed using a Dionex Ultimate 3000 UHPLC system (HPG 3200 RS binary pump, WPS-3000 TRS autosampler, TCC-3000 RS thermostatic column compartment and DAD detector, interfaced with an H-ESI II probe and an LTQ Velos pro linear ion trap mass spectrometer (Thermo Fisher Scientific, Germering, Germany)). Separation of phenolic compounds was performed using an Acclaim 120 C18 column (120 Å, particle size 3 µm, 3 × 150 mm; Thermo Fisher Scientific, Waltham, MA, USA), maintained at 30 °C. The mobile phase consisted of (A) water/formic acid 0.1% (99.9:0.1, *v*/*v*) and (B) acetonitrile/formic acid 0.1% (99.9:0.1, *v*/*v*), at a constant flow rate of 0.55 mL min^−1^. The gradient program for solvent A was as follows: 0–1 min, 94% isocratic; 1–26 min, increase to 55%; 26–33 min, decrease to 30%; 33–36 min, 30% isocratic; 36–55 min, increase to 94%. Then, equilibration was achieved under the initial conditions for 5 min. Detection was performed at 330 nm. The MS parameter conditions were as follows: capillary temperature, 320 °C; source heater temperature, 280 °C; nebulizer gas, N2; sheath gas flow, 30 arbitrary units; auxiliary gas flow, 7 arbitrary units; capillary voltage, −2800 V, S-Lens RF level 60%. Data were acquired in negative ionization mode. Samples were analyzed with two methods: a full-scan method from 150 to 1200 *m*/*z* and a data-dependent experiment to collect MS/MS data. The data-dependent settings included a full scan from 150 to 1200, an activation level of 65,000 counts, an isolation width of 2 Da, a default charge state of 2, and CID energy of 35. Phenolic compounds were identified using UV spectra, molecular ion, the respective fragments produced in MS2 and retention time in accordance with the literature [21].

### 2.4. Semi-Quantification of Phenolic Compounds by Ultra-High-Performance Liquid Chromatographic–Diode Array (UHPLC-DAD)

Semi-quantification of the phenolic compounds was performed using a Dionex Ultimate 3000 UHPLC system (HPG 3200 RS binary pump, WPS-3000 TRS autosampler, TCC-3000 RS column thermostat compartment and DAD detector; Thermo Fisher Scientific, Germering, Germany). Phenolic compound separation was performed using an Acclaim 120 C18 column (120 Å, 3 µm particle size, 3 × 150 mm; Thermo Fisher Scientific, Waltham, MA, USA), which was maintained at 30 °C. The mobile phase consisted of (A) water/formic acid 0.1% (99.9:0.1, *v*/*v*) and (B) acetonitrile/formic acid 0.1% (99.9:0.1, *v*/*v*), with a constant flow rate of 0.55 mL min^−1^. The gradient program for solvent A was as follows: 0–1 min, isocratic at 94%; 1–26 min, increase to 55%; 26–33 min, decrease to 30%; 33–36 min, isocratic at 30%; 36–55 min, increase to 94%. Then, equilibration was achieved under the initial conditions for 5 min. Detection was conducted at 330 nm, and semi-quantitative analysis was carried out using the external standard method, based on a calibration curve obtained by injecting 5-*O*-caffeoylquinic acid at concentrations ranging from 1000 mg L^−1^ to 7.8125 mg L^−1^ (R^2^ = 0.9994–0.9997). Values are expressed as mg 5-*O*-caffeoylquinic acid equivalents/L.

### 2.5. Cell Culture Conditions and Treatments

In vitro tests on Caco-2 human colorectal cancer cells (ICLC HTL 97023) and human colorectal adenocarcinoma HT29 cells (ICLC HTL99026) both obtained from Interlab Cell Line Collection (Genoa, Italy) were performed. Both cell lines were maintained in D-MEM supplemented with 100 U/mL penicillin, 100 μg/mL streptomycin, glutamine (2 mM), and 10% fetal bovine serum (FBS, UE approved origin) (all reagents were purchased from Life Technologies-Invitrogen, Milan, Italy). Both cell cultures were grown at 37 °C in a humidified incubator containing 5% CO_2_ and expanded in tissue culture flasks (75 cm^2^, BD Biosciences, Milan, Italy), changing the medium every two days. The cells were seeded in six-well cell culture plates and 96-multiwell plates, cultured to reach 80% confluency, and then submitted to subsequent treatments.

For the cytotoxicity test, Caco-2 and HT29 cells were exposed to four samples of artichoke aqueous extracts (BriB, VaMB, LMTB, CAPB) at various concentrations (0.1, 0.25, 0.5, 0.8, 1, 2, 3 and 4 mg/mL) of each extract for 24 and 48 h. For cell apoptosis, we considered 2 and 3 mg/mL concentrations for all extracts and 24 h as the optimized time. Untreated cells were used as control.

### 2.6. MTT Test

Cell viability was analyzed by using the 3-(4,5-dimethylthiazol-2-yl)-2-5-diphenyltetrazolium bromide (MTT) (Sigma-Aldrich, St. Louis, MO, USA) assay. Briefly, Caco-2 and HT29 cells were seeded in a 96-well plate at a density of 8 × 10^3^ cells/well. The next day, the cells were treated with different concentrations (see above) of each extract for 24 and 48 h. After the incubation period, culture media were carefully removed, and 100 μL of 0.5 mg/mL MTT in cell culture medium was added to each well. After incubation for 4 h at 37 °C in a humidified atmosphere with 5% CO_2_, the formazan crystals formed were dissolved by adding 150 μL of Dimethylsulfoxide (DMSO) to each well for 20 min under stirring. Cell viability was measured by reading the absorbance (540 nm) on a SPECTROStar Nano Absorbance reader (BMG Labtech, Ortenberg, Germania). The background absorbance of multiwell plates was measured at 690 nm and subtracted from the 540 nm measurement. Data were converted to percentages, taking the control as 100%, and expressed as the average percentage ± SD.

### 2.7. Morphological Evaluation Using DAPI Staining

Cellular morphological alterations due to apoptosis and induced by artichoke aqueous extracts in Caco-2 and HT29 cells were assessed via 4,6-diamidino-2-phenylindole (DAPI) (Sigma-Aldrich, Milan, Italy) staining. Both cell types, seeded in 4-well Nunc plates at 5 × 10^4^ cells per well, were treated with each extract at a concentration of 2–3 mg/mL for 24 h, and subsequently fixed with 4% paraformaldehyde. The fixed cells were washed 3 times with PBS and then permeabilized with 0.1% (*v*/*v*) Triton X-100 (Sigma-Aldrich, Milan, Italy). The cells were stained with 1 µg/mL DAPI dye for 30 min in the dark. To remove excess dye, the cells were washed with PBS, and nuclear morphological changes were visualized by the EVOS M5000 Imaging System fluorescence microscope (ThermoFisher Scientific, Milan, Italy).

### 2.8. Apoptosis Analysis by Annexin V-CY3 Staining

To detect cell apoptosis, the Annexin V-Cy3 (AnnCy3) Apoptosis Detection kit (Sigma Aldrich, Milan) was used. Annexin V has a high binding affinity for phospholipid phosphatidylserine, which normally, in healthy cells, is found on the cytoplasmic interface of the plasma membrane but can translocate to the outer membrane in apoptotic cells. Under this condition, phosphatidylserine is free to bind to AnnCy3. Briefly, Caco-2 and HT29 cells were seeded into 4-well Nunc plates at 5 × 10^4^ cells per well at 37 °C in a humidified atmosphere with 5% CO_2_. The next day, the medium was replaced with complete medium with the addition of each artichoke extract at concentrations of 2–3 mg/mL. After 24 h of incubation, cell cultures were washed with PBS followed by washes with binding buffer and then were incubated with double-label staining solution, AnnCy3 and 6-Carboxyfluorescein diacetate (6-CFDA) for 10 min at room temperature in the dark. Subsequently, the cell cultures were washed five times with binding buffer to remove excess label, and apoptotic cells were detected by using the EVOS M5000 Imaging System fluorescence microscope (ThermoFisher Scientific, Milan, Italy). This kit allows for the simultaneous observation of three different cell populations: live cells exhibit green fluorescence (6-CFDA+) (filter 455 nm), necrotic cells exhibit red fluorescence (AnnCy3+) (filter 530 nm), and apoptotic cells stain with both AnnCy3(red) and 6-CFDA (green). The percentage of apoptotic cells was determined by counting at least100 cells.

### 2.9. Protein Extraction and Western Blot Analysis

Caco-2 and HT29 cells were seeded at a density of 5 × 10^5^ per well in 6-well plates and cultured for 24 h. Then, cells were treated with 2–3 mg/mL of each extract for 24 h to evaluate the level of expression of the indicated proteins. After treatment, cells were gently detached from the culture plates by scraping and collected after centrifugation at 2000 rpm for 10 min at 4 °C. Subsequently the obtained cell pellets were lysed for 30 min on ice with lysis buffer [1% Triton X-100, 20 mM Tris–HCl, 137 mM NaCl, 10% glycerol, 2 mM EDTA, 1 mM phenylmethylsulfonyl fluoride (PMSF), 20 μM leupeptin hemisulfate salt, 0.2 U/mL aprotinin (Sigma-Aldrich)]. The cell lysates were centrifuged at 14,000 rpm for 20 min at 4 °C to remove insoluble debris after undergoing ten freeze–thaw cycles. The protein concentration in the supernatant was spectrophotometrically determined with Bradford’s protein assay. Briefly, protein samples were diluted with 2× Laemmli sample buffer and β-mercaptoethanol following the manufacturer’s instructions (Bio-Rad Laboratories, Hercules, CA, USA) and then boiled for 5 min at 95 °C. Proteins (25 μg/lane) and pre-stained standards (Bio-Rad Laboratories, Hercules, CA, USA) were loaded on 4–15% Mini-PROTEAN^®^ TGX™ SDS precast polyacrylamide gels (Bio-Rad Laboratories, Hercules, CA, USA) and subjected to SDS-PAGE in order to separate proteins by molecular weight under denaturing conditions, allowing for subsequent identification and relative quantification of specific target proteins by immunoblotting. Electrophoresis was performed using a Mini-PROTEAN^®^ Tetra Cell system (Bio-Rad Laboratories, Hercules, CA, USA) at a constant voltage of 200 V for approximately 30–35 min in Tris-glycine-SDS running buffer. After electrophoresis, proteins were transferred to a nitrocellulose membrane, blocked in EveryBlot Blocking Buffer (Bio-Rad Laboratories, Hercules, CA, USA) for 5 min at room temperature, and then washed 3 times with 0.1% Tween 20-TBS (T-TBS). Next, membranes were probed overnight at 4 °C with primary mouse monoclonal antibody (moAb) anti-β-actin, moAb anti-caspase-3, moAb anti-caspase-9, moAb anti-Bcl-2, and moAb anti-Bax (all from Santa Cruz Biotechnology, Inc., Milan, Italy), all used at a 1:500 dilution. The blots were washed with T-TBS (for 20 min, 3 times) and then exposed for 1 h at room temperature on a shaker with a secondary antibody anti-mouse or anti-goat IgG conjugated to horseradish peroxidase (HRP) (1:10,000, Bentham, Milan, Italy). After three washes with 0.1% T-PBS, immunoreactive bands were acquired using a ChemiDoc™ Touch Imaging System (Bio-Rad Laboratories, Hercules, CA, USA). The optical density of each band was normalized to the corresponding β-actin level and expressed as the mean ± SD. A chemiluminescent method (Bio-Rad Laboratories, Hercules, CA, USA) was used to visualize the bands.

### 2.10. Statistical Analysis

All data are given as the mean ± standard deviation (SD) of triplicate measurements from five independent experiments. Statistically significant differences (*p* < 0.05) were evaluated using Student’s *t*-tests and analysis of variance (one-way ANOVA). Statistical analysis of the data was performed using Minitab statistical software version 21.1.0 (Minitab Inc., State College, PA, USA).

## 3. Results

### 3.1. Total Phenol Content and Identification and Semi-Quantitative Analysis of Phenolic Compounds

The identification of phenolic compounds was first performed by UHPLC-MS/MS analysis. Based on the highest TPC values, a representative UHPLC chromatogram of VaMB extract, with the identified compounds labeled according to Table 1, is provided in the Supplementary Material (Figure S1), together with the corresponding mass spectra of the main identified compounds. Phenolic compounds were identified using UV spectra, molecular ions and the respective fragments produced in MS2. The figure shows the chromatographic profiles of the analyzed sample. By comparing the molecular ions and the corresponding fragments produced in MS2 with the literature [21], eight phenolic compounds were identified: 5-*O*-caffeoylquinic acid, 353 [M-H]^−^ (*m*/*z*); luteolin 7-*O*-rutinoside, 593 [M-H]^−^ (*m*/*z*); luteolin 7-*O*-glucoside, 447 [M-H]^−^ (*m*/*z*); luteolin 7-*O*-glucuronide, 461 [M-H]^−^ (*m*/*z*); apigenin 7-*O*-rutinoside, 577 [M-H]^−^ (*m*/*z*); 3,5-di-*O*-caffeoylquinic acid, 515 [M-H]^−^ (*m*/*z*); 3,4-di-*O*-caffeoylquinic acid, 515 [M-H]^−^ (*m*/*z*); apigenin 7-*O*-glucuronide, 445 [M-H]^−^ (*m*/*z*).

Table 1 shows the TPC and the semi-quantitative profile of the main phenolic compounds identified by UHPLC-DAD analysis. Significant differences were observed between the four artichoke varieties in terms of both TPC and phenolic composition. Specifically, VaMB exhibited the highest TPC among the extracts tested (269.51 ± 5.12 mg GAE/L), followed by LMTB (254.48 ± 2.91 mg GAE/L) and BriB (206.24 ± 5.35 mg GAE/L). In contrast, CAPB exhibited significantly lower values (122.70 ± 3.79 mg GAE/L) (*p* < 0.05). These results confirm the greater phenolic richness of the local Apulian varieties compared to the hybrid cultivar (CAPB).

UHPLC-DAD analysis revealed that hydroxycinnamic acids were the predominant phenolic class in all samples. Specifically, 5-*O*-caffeoylquinic acid was significantly more abundant in VaMB (19.30 ± 2.77 mg/L) than in the other varieties. 3,5-di-*O*-caffeoylquinic acid was predominantly detected in VaMB, whereas the highest concentration of 3,4-di-*O*-caffeoylquinic acid (14.27 ± 0.38 mg/L) was found in LMTB. Flavonoid derivatives were present at lower concentrations than hydroxycinnamic acids. Luteolin-7-*O*-glucoside was particularly abundant in BriB and CAPB, while apigenin-7-*O*-rutinoside was significantly higher in VaMB. The quantified total phenolic content reflected the TPC trend, with VaMB showing the highest concentration of phenolic compounds, followed by LMTB. However, BriB and CAPB showed significantly lower values.

### 3.2. Effects of Artichoke Extracts on Intestinal Cell Viability

**Figure 1:**
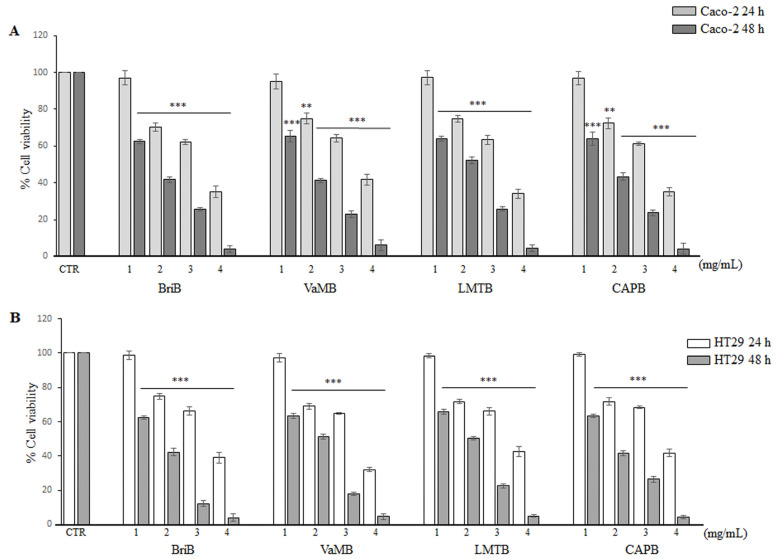
The effects of artichoke extracts on cell viability (%). Caco-2 (**A**) and HT29 (**B**) cells were exposed to different artichoke extracts (BriB, VaMB, LMTB, CAPB) at concentrations ranging from 1 to 4 mg/mL. Cell viability was assessed at 24 and 48 h via the MTT assay. Untreated cells represent the control (CTR). Data are presented as the mean ± SD of five independent experiments (*** *p* < 0.001 and ** *p* < 0.01 vs. CTR).

The effects of four artichoke extract samples (BriB, VaMB, LMTB, and CAPB) on the viability of Caco-2 (Figure 1A) and HT29 (Figure 1B) cells were evaluated using an MTT assay. Both cell lines were exposed to different concentrations of each extract (1, 2, 3, and 4 mg/mL) for 24 and 48 h. As shown in Figure 1, exposure to 1 mg/mL of the extracts for 24 h did not significantly affect cell viability in either cell line compared with the untreated control. In contrast, after 48 h of treatment, the same concentration significantly reduced cell viability in both cell lines compared to untreated controls. All the other concentrations of each extract (2, 3, and 4 mg/mL) significantly reduced cell viability in both Caco-2 and HT29 cells. Furthermore, this reduction was concentration- and time-dependent. This effect was markedly pronounced after 48 h of exposure, reaching viability levels close to zero at the highest concentration (4 mg/mL). Cells treated with concentrations lower than 1 mg/mL of each extract showed no significant decrease in cell viability when compared to untreated cells (control). Interestingly, both Caco-2 and HT29 showed a similar response profile towards the extracts used, indicating similar antiproliferative activity of the tested extracts on both cell lines. However, slight variations in sensitivity may be observed at specific intermediate concentrations (2–3 mg/mL) after 24 h of treatment. Based on these results, concentrations of 2–3 mg/mL at 24 h were selected as the optimal conditions for subsequent apoptosis studies (as described in Section 2.4).

### 3.3. Artichoke Extracts Promote Apoptosis in Intestinal Cells

#### 3.3.1. Cell Morphology Analysis

**Figure 2:**
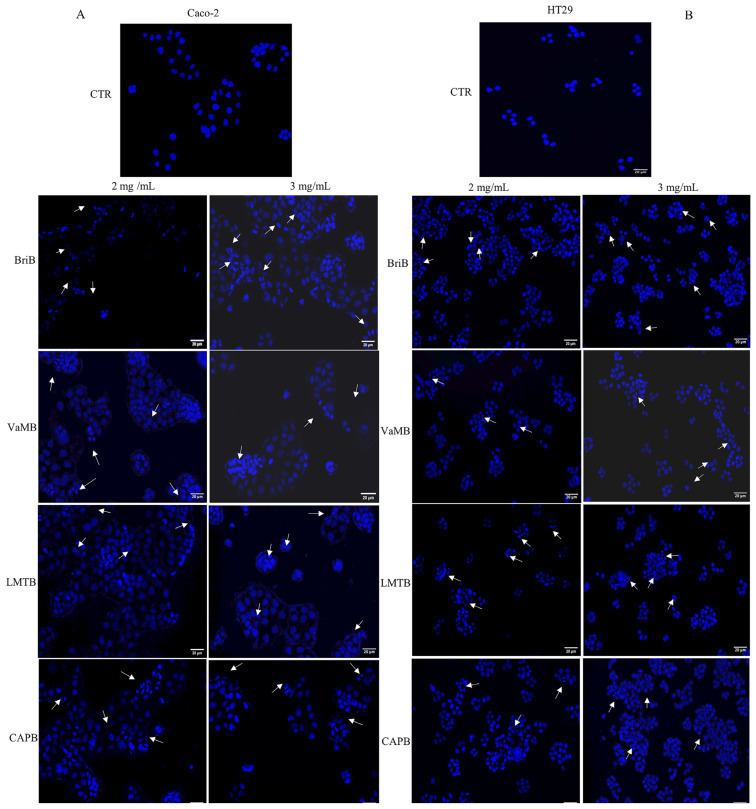
Nuclear morphological modification induced by artichoke extracts in Caco-2 and HT29 cell lines. DAPI-stained Caco-2 (**A**) and HT29 (**B**) cells. Cells were treated with 4 types of extracts at concentrations of 2–3 mg/mL of each extract (BriB, VaMB, LMTB and CAPB) for 24 h. After 24 h, cells were stained with DAPI dye and then visualized using the EVOS M5000 Imaging System fluorescence Microscope. Cells treated with extracts at both concentrations showed typical characteristics of apoptotic cells such as nuclear fragmentation and chromatin condensation (white arrows) and non-uniform and brighter nuclei. No morphological changes were observed in the control cell lines. The scale bar corresponds to 20 µm.

Based on the MTT assay results (Section 3.2) and the treatment conditions described in Section 2.4, Caco-2 and HT29 cells were treated with 2–3 mg/mL of each artichoke extract (BriB, VaMB, LMTB, and CAPB) to assess apoptosis induction. A treatment time of 24 h was selected for subsequent nuclear morphology analysis, with morphological changes being assessed by nuclear staining with DAPI. Analysis of nuclear integrity by DAPI staining revealed significant differences between controls and the treated samples. Our study highlighted that all the extracts tested at the concentrations used (2–3 mg/mL) induced apoptosis in both cell lines (Figure 2), while no morphological changes were observed in untreated cells (control). Specifically, Caco-2 (Figure 2A) and HT29 cells (Figure 2B) treated with artichoke extracts showed non-uniform and brighter nuclei, chromatin condensation, and nuclear fragmentation (white arrows), whereas untreated cells showed nuclei that appeared more homogeneously. In Caco-2 cells, BriB and CAPB induced the highest degree of nuclear fragmentation at both concentrations tested, followed by VaMB.

In HT29 cells, BriB and LMTB induced the highest degree of nuclear fragmentation at both concentrations tested, followed by CAPB.

Overall, these morphological observations support the ability of the extracts to induce apoptosis in both colorectal cancer cell lines.

**Figure 3:**
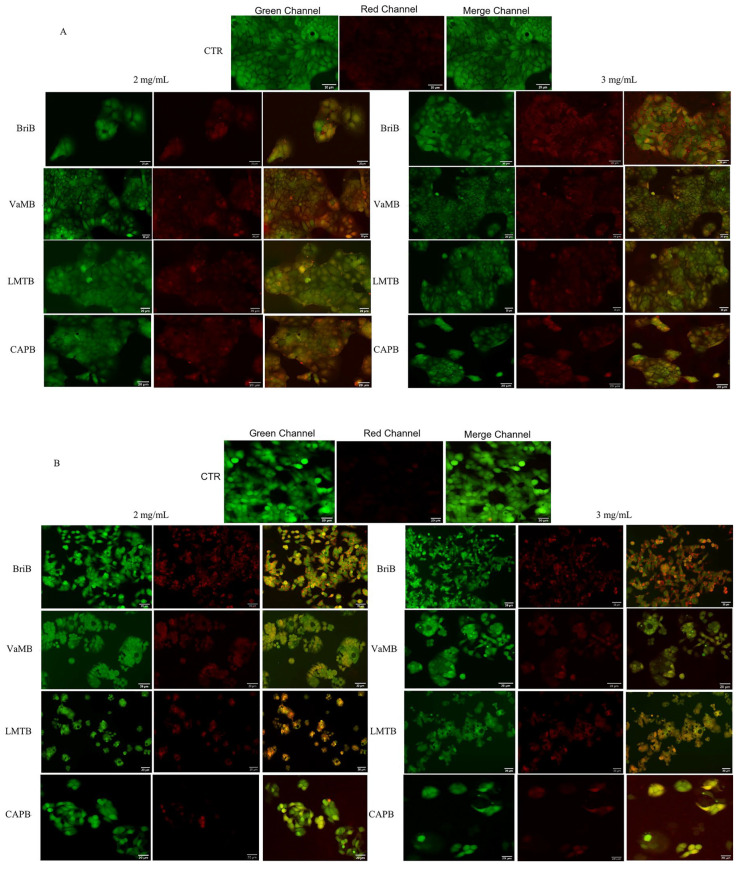
The induction of apoptosis mediated by artichoke extracts. AnnCy3/6-CFDA double-staining assay in Caco-2 (**A**) and HT29 (**B**) cell lines. Cells were treated for 24 h with 4 types of extracts (BriB, VaMB, LMTB and CAPB) at concentrations of 2–3 mg/mL of each extract. After incubation, cells were stained with AnnCy3 and 6-CFDA and analyzed using the EVOS M5000 Imaging System fluorescence Microscope. Viable cells showed green fluorescence, necrotic cells showed red fluorescence and apoptotic cells showed yellow/orange fluorescence. The images are shown as representative of 3 independent experiments. The scale bar corresponds to 20 µm.

**Figure 4:**
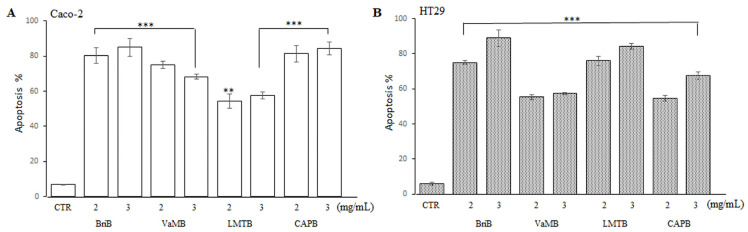
The percentage of apoptotic cells assessed by using the AnnCy3/6-CFDA double-staining assay. For each extract, concentrations of 2–3 mg/mL were used, and apoptotic cells were counted in 4–5 randomly selected fields. The percentage of apoptotic Caco-2 (**A**) and HT29 (**B**) cells was determined by counting at least 100 cells. Values are presented as the mean ± SD of three independent experiments (*** *p* < 0.001 and ** *p* < 0.01 vs. CTR).

To further confirm that the extracts promoted cell apoptosis, Caco-2 (Figure 3A) and HT29 (Figure 3B) cells, after 24 h of treatment with the extracts, were stained with a double label, AnnCy3/6-CFDA, and apoptosis induction was assessed by fluorescence microscopy analysis. Figure 3 shows three different cell populations after treatment with each extract at concentrations of 2–3 mg/mL. Viable cells, positive for 6-CFDA, showed green fluorescence; necrotic cells, positive for AnnCy3, exhibited red fluorescence; and apoptotic cells (AnnCy3+/6-CFDA+) were stained yellow/orange. The percentage of apoptotic cells was determined after counting through microscopic observation of at least 100 cells in four to five different microscopic fields for each extract at the two concentrations used. As Figure 4 shows, all four extracts, with varying percentages, caused apoptotic cell death in the two cell lines. Precisely, in Caco-2 cells (Figure 4A), BriB and CAPB extracts were able to cause an apoptosis rate higher than 80% at both concentrations used, while VaMB extract caused an apoptotic rate ranging from 70 to 75% at both concentrations used (2–3 mg/mL). Notably, only the LMTB extract caused an apoptosis rate below 60% at both concentrations used. In HT29 cells (Figure 4B), BriB extract caused the same rate of apoptosis observed in Caco-2 cells, while VaMB and CAPB extracts caused an apoptotic rate ranging from 55 to 65% at concentrations of 2 and 3 mg/mL, respectively. Finally, LMTB extract caused a higher apoptotic rate (75–85%) in HT29 cells than that observed in Caco-2 cells.

#### 3.3.2. Expression Levels of Apoptotic Proteins

**Figure 5:**
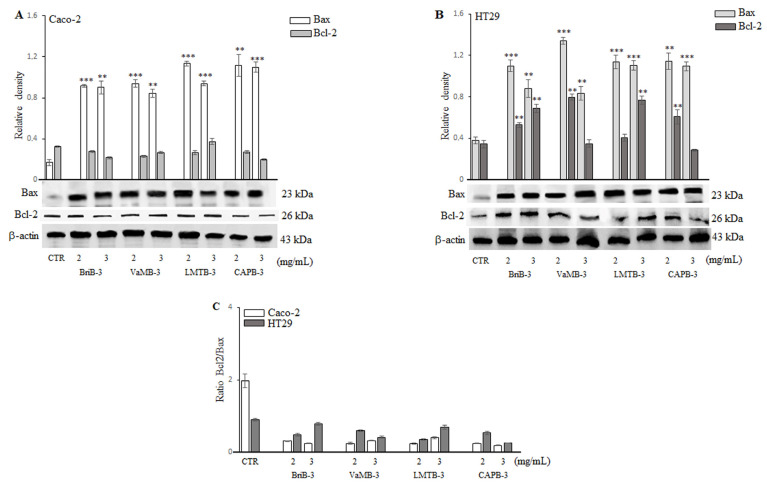
The effects of artichoke extracts on the expression levels of Bax and Bcl-2 proteins. Immunoblotting detection of Bax and Bcl-2 in Caco-2 (**A**) and HT29 (**B**) cells treated with 2 and 3 mg/mL of each artichoke extract (BriB, VaMB, LMTB, CAPB) for 24 h. The Bcl-2/Bax ratio for both cell types is shown in panel (**C**). Untreated cells represent the control (CTR). Densitometric analysis values are presented as arbitrary units after normalization against β-actin. The results are shown as the mean ± SD of five independent experiments (*** *p* < 0.001 and ** *p* < 0.01 vs. CTR).

**Figure 6:**
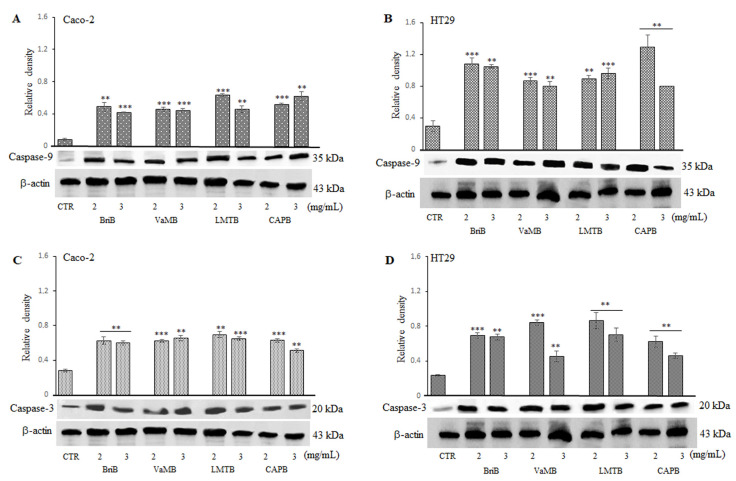
The effects of artichoke extracts on the expression levels of caspase-9 and caspase-3 apoptotic proteins. Western blotting detection of caspase-9 and caspase-3 in Caco-2 (**A**,**C**) and HT29 (**B**,**D**) cells treated for 24 h with 2 and 3 mg/mL of each artichoke extract (BriB, VaMB, LMTB, CAPB). Untreated cells were used as the control (CTR). Densitometric analysis values are presented as arbitrary units after normalization against β-actin. The results are shown as the mean ± SD of five independent experiments (*** *p* < 0.001 and ** *p* < 0.01 vs. CTR).

The effect of artichoke extracts on the expression levels of apoptotic proteins in Caco-2 and HT29 tumor cells was further investigated at the same concentrations (2–3 mg/mL, 24 h) established in Section 2.4. Specifically, the impact of each extract on the expression levels of the pro-apoptotic protein Bax, the anti-apoptotic Bcl-2, the initiator caspase-9, and the executioner caspase-3 was evaluated. The four artichoke extracts at the tested concentrations (2 and 3 mg/mL) resulted in a significant increase (*p* < 0.01 and *p* < 0.001) in Bax protein expression levels in both Caco-2 and HT29 cells compared to control cells (Figure 5A,B). Furthermore, Caco-2 cells’ expression of the anti-apoptotic protein Bcl-2 was significantly lower than that shown by untreated cells (Figure 5A). The Bcl-2/Bax expression ratio in these cells was also greatly decreased (Figure 5C). Conversely, although not all concentrations of the four extracts caused a significant increase in Bcl-2 expression levels in HT29 cells (Figure 5B), they all led to a strong reduction in the Bcl-2/Bax ratio overall (Figure 5C). Figure 6 shows that artichoke extracts, at both concentrations, resulted in significant high expression of caspase-9 (Figure 6A,B) and caspase-3 (Figure 6C,D) in both cell lines. In this respect, Table 2 and Table 3 report the relative significance of the expression levels of apoptotic proteins in Caco-2 and HT29 cells exposed to the various groups of extracts at the indicated concentrations. In Caco-2 cells, LMTB increased caspase-9 expression at 2 mg/mL and caspase-3 expression at both concentrations. In HT29 cells, BriB increased caspase-9 expression at both concentrations, whereas the 3 mg/mL concentration was more effective in enhancing caspase-3 expression. However, all four extracts were able to determine an increase in expression of both caspase-9 and caspase-3. Overall, these results suggest that our artichoke extracts induce apoptotic cell death in Caco-2 and HT29 cell lines.

## 4. Discussion

Dietary polyphenols are attracting increasing attention for their potential role in preventing and modulating CRC, largely due to their ability to interfere with pathways related to oxidative stress, inflammation and apoptosis [22,23,24]. Specifically, hydroxycinnamic acids and flavonoids derived from plants have been shown to influence mitochondrial function and the balance between pro- and anti-apoptotic proteins, thereby contributing to the regulation of tumor cell survival [25]. However, the biological efficacy of plant extracts depends heavily on their qualitative and quantitative phytochemical composition, which in turn is influenced by the cultivar, geographic origin, and processing conditions [14,20]. Among plant species, artichoke is one of the most extensively investigated due to its richness in bioactive components. In recent years, considerable attention has been given to artichoke by-products (leaves, bracts, and stems) recognized as valuable sources of phenolic compounds and other health-promoting molecules [15,26].

Based on these findings, our previous study provided a thorough characterization of extracts obtained from artichoke bracts of various cultivars grown in Apulia, as well as hybrids [20]. This study revealed significant differences in phenolic composition depending on the cultivar, demonstrating that certain local varieties were particularly rich in caffeoylquinic acid derivatives and flavonoids and exhibited superior antioxidant and prebiotic properties. Building on these findings, the present study examined the impact of artichoke by-products from the more promising cultivars tested (one hybrid, CAPB, and three local varieties, BriB, VaMB and LMTB) on apoptosis mechanisms in colon cancer cells by evaluating their phenolic compositions. UHPLC-DAD profiling of the samples confirmed the variability in composition and further supported the distinctive phenolic profiles of the selected cultivars. Specifically, VaMB and LMTB were found to be the richest in phenolic compounds, while the hybrid cultivar CAPB exhibited relatively lower values, consistent with previous observations [20]. It is important to note that the quantified total phenolic compounds were only a small part of the TPC. This suggests that there are other phenolic compounds which were not included in the UHPLC-DAD analysis and highlights the complexity of the phytochemical matrix, in line with other studies [27]. Moreover, Folin–Ciocalteu reagent is not entirely specific for phenolic compounds, as it can also react with other reducing substances, such as ascorbic acid, reducing sugars, and other antioxidant molecules. Consequently, TPC values may reflect the overall reducing capacity of the extracts rather than exclusively reflecting their phenolic content, which may further contribute to the differences observed between Folin–Ciocalteu and UHPLC-DAD results.

Given the well-documented pro-apoptotic activity of caffeoylquinic acids and flavonoids, which were more abundant in VaMB and LMTB, their potential biological effects in colon cancer cells were evaluated.

In this work, we analyzed the cytotoxic and pro-apoptotic effects of the different artichoke by-product extracts in Caco-2 and HT29 human colorectal adenocarcinoma cell lines. We observed that all four extracts reduced cell viability in both cell lines in a concentration- and time-dependent manner. For all extracts, concentrations ≥2 mg/mL significantly reduced cell viability at both time points, with viability nearly abolished at 4 mg/mL after 48 h of treatment. These results are in line with previous studies reporting cytotoxic effects of artichoke-derived extracts in other tumor models such as breast cancer and oral squamous carcinoma [28,29,30], as well as on different human colon cancer cell lines [31,32]. Additionally, both Caco-2 and HT29 cells showed a similar sensitivity profile, thus suggesting that the antiproliferative effects shown are not strictly cell line-specific. However slight differences in sensitivity were evident at the intermediate concentrations tested (2–3 mg/mL), possibly reflecting the distinct morphological, functional, and differentiation characteristics of the two different cell lines, with Caco-2 cells primarily being used as a model for the absorptive enterocytes of the small intestine, while HT29 cells are used to model mucus-secreting goblet cells [33,34]. Comparative analysis of the four artichoke extracts studied reveals a critical efficacy threshold identified between 2 and 3 mg/mL. Therefore, the biological activity shown by the extracts seems strongly correlated with the total phenolic content (TPC). This observation is supported by other studies conducted on intestinal cells where extracts with a high phenolic content derived from another matrix were used. Indeed, it has been demonstrated that phenol-rich extracts prepared from biotransformed grape pomace can determine an antiproliferative effect in the colorectal cancer cell lines Caco-2 and SW620 [35] as well as in HT29 and SW480 [36]. This suggests the presence of possible bioactive compounds in the extracts obtained from the waste products, including polyphenols and flavonoids, which could be responsible for inhibiting the growth of tumor cells.

To test whether the reduction in cell viability was associated with the induction of apoptosis, we assessed nuclear morphology using DAPI nuclear staining. Cells treated with all four different artichoke extracts at 2–3 mg/mL for 24 h showed clear signs of apoptosis, such as brighter nuclei and nuclear fragmentation, while untreated control cells maintained intact and homogeneous nuclei. These observations are consistent with the morphological features of apoptotic cell death that are well documented in the literature [1,37,38]. Interestingly, some differences in the intensity of nuclear modifications were observed between the four extracts tested. BriB and CAPB extracts in Caco-2 cells and BriB and LMTB extracts in HT29 cells showed a greater ability to induce nuclear fragmentation at both concentrations used.

Furthermore, we also analyzed the apoptosis rate in both cell lines using a double label, AnnCy3/6-CFDA. Spontaneous apoptosis was barely detectable in untreated cells, while all of the different extracts used in the treatments significantly increased the number of apoptotic cells. Specifically, in both colon cancer cell lines used, the BriB extract was, once again, the most effective in inducing apoptosis, especially at a concentration of 3 mg/mL. In Caco-2 cells, in addition to BriB, the CAPB extract was also particularly effective in inducing apoptosis, followed by the VaMB extract, while the apoptotic rate determined by the LMTB extract was significantly lower. This trend is different, however, in HT29 cells where the BriB extract and the LMBT extract were able to induce a significantly higher percentage of apoptosis than those obtained from VaMB and CAPB, thus confirming the results obtained from the analysis of nuclear morphology in the cell lines treated with the extracts under examination.

In this context, although VaMB exhibited the highest total polyphenol content, it induced a lower rate of apoptosis than BriB, particularly in HT29 cells. Conversely, in Caco-2 cells, LMTB appeared to be the least effective in inducing apoptosis. Notably, similarly to VaMB, this extract showed a high total polyphenol content compared with the other extracts, but a lower flavonoid content.

Based on these observations, it can therefore be hypothesized that the differences in cellular response in terms of apoptosis induction and in relation to treatment with the single extracts may be determined by the different compositions of the bioactive compounds contained in each extract.

Although VaMB and LMTB are the extracts richer in caffeoylquinic acids and with the highest TPC, the BriB extract exhibits the highest concentration of luteolin-7-*O*-glucoside and apigenin-7-*O*-glucuronide. Luteolin has been reported to be crucial in cancer cell organization by acting on several mechanisms of tumor development and progression, including cell cycle arrest induction and apoptosis promotion in different cancer types [39,40]. Luteolin’s anticancer effects have also been observed in human colon cancer cells. It was, in fact, demonstrated that luteolin promotes apoptotic cell death in both HT29 and SNU-407 colon cancer cells [41]. Some authors report that luteolin-7-*O*-glucoside appears to be very effective in inducing apoptosis in various types of tumors with reference to colon cancer [42,43]. In addition to luteolin, apigenin, a flavonoid found naturally in various plants, has gained considerable attention in cancer research for some of its properties, including its pro-apoptotic properties [44]. Apigenin 7-*O*-glucuronide, a major metabolite of apigenin, shows greater stability, demonstrating significant potential as a natural drug offering superior efficacy to apigenin [45,46]. Furthermore, it was also observed that extracts particularly rich in apigenin obtained from artichoke belonging to the cultivar “*Carciofo di Procida*” showed significant cytotoxic activity against SH-SY5Y neuroblastoma and Caco-2 colorectal adenocarcinoma cell lines [47]. Moreover, 5-fluorouracil is currently one of the most effective chemotherapeutic agents for colorectal cancer; however, its clinical use is limited by drug resistance. In this regard, apigenin alone has been reported to have modest antitumor effects on the human colon cancer cell lines HCT116 and HT29, but when administered with 5-fluorouracil, it increases the degree of cell apoptosis and is therefore able to enhance its efficacy by reducing acquired resistance to chemotherapy [48]. All these observations, therefore, suggest that the antitumor activity shown by the different artichoke extracts derived from by-products used in our experiments could depend not only on the TPC, but also on the specific synergy between the compounds contained in each extract. In this regard, it is well known that the combination of different phytochemicals can produce additive or synergistic antitumor effects, with an overall efficacy greater than the combined effect of the individual contributions. In particular, treatment of primary and highly metastatic breast cancer cell lines with a combination of six different phytochemicals causes a significant reduction in cell proliferation, motility, and invasion, with concomitant induction of apoptosis [49,50]. It has been specifically shown, for example, that luteolin and curcumin synergistically inhibit the growth of colon cancer cells at concentrations where each compound alone has no significant effect [51], and that combinations of polyphenols belonging to different chemical classes can cooperatively modulate apoptotic pathways in colon cancer cells [13,52]. This could explain the greater efficacy of BriB in inducing apoptosis in the two colon cancer cell lines used in our study, despite its lower TPC than VaMB and LMTB.

In the apoptotic process, Bax and Bcl-2 proteins play a key role in regulating mitochondrial membrane permeability. An upregulation of Bax determines the increased permeabilization of the membrane, ensuring the release of apoptotic effectors which, in turn, through a sequence of biochemical events, lead to the activation of cytoplasmic caspases. Conversely, upregulation of Bcl-2 leads to the inhibition of cellular apoptosis [53,54]. Our by-products from artichoke extracts possess a marked ability to modulate the protein network linked to cell survival. The most significant result emerges from the Bcl-2/Bax ratio. In control cells, the ratio is clearly unbalanced towards Bcl-2, ensuring tumor cell survival. Following exposure to the extracts, a significant decrease in this ratio is observed in both Caco-2 and HT29. Overall, the decrease in the Bcl-2/Bax ratio observed in all treatments, with slight differences between the various extracts, confirms that the phytocomplex contained in the extracts obtained from artichoke waste acts as a potent cellular modulator, irreversibly directing tumor cells toward programmed death through the mitochondrial pathway. Our observations are consistent with others reported in the literature. The extracts obtained from the edible parts and leaves of fresh artichoke are reported to decrease the expression of anti-apoptotic Bcl-2 proteins, increase pro-apoptotic Bax proteins, and trigger mitochondrial apoptosis in several tumor cell lines including colorectal cancer cell lines [29,55,56]. Accordingly, our results suggest that consistent upregulation of Bax and slight downregulation of Bcl2 may be the molecular mechanisms through which our extracts induce the intrinsic apoptotic pathway. Therefore, to deepen the mechanism involved in the induction of apoptosis in colon cancer cells treated with the extracts of our interest, we evaluated the modulation of caspase-9 and caspase-3. These are key proteolytic enzymes in the process of programmed cell death, acting in a sequential cascade. Caspase-9 is known to be an intrinsically activated initiator, which in turn activates caspase-3, the effector caspase responsible for the final degradation of cellular proteins [57]. All of the different artichoke extracts used in our experiments were able to significantly activate both caspase-9 and caspase-3 in both cell lines when compared to control conditions. This observation is in line with a previous in vitro study in which an increase in the expression of the two caspases in the leukemic cell line HL-60 was demonstrated [58]. Moreover, our results are also in line with a recent in vivo study reporting that the upregulation of the caspase-3 and caspase 9 pathways may be the mechanisms behind the antitumor actions of artichoke extracts in a mouse model of hepatocellular carcinoma [59]. Interestingly, moreover, our data highlight that the upregulation of caspase-9 and caspase-3 in Caco-2 cells appears to be rather homogeneous, while in HT29 cells the upregulation of caspases, particularly caspase-9, appears stronger than what was observed in Caco-2 cells. Thus, although our extracts are effective in both cell models, the HT29 cell line appears to be a more responsive model, particularly regarding the initiation of the caspase cascade through the action of caspase-9.

Our observation is supported by a study in the literature that investigated the antitumoral effects of grape pomace and grape seed extracts on Caco-2 and HT29 cells which described that Caco-2 responds more to crude extracts while HT29 is more sensitive to purified fractions, depending mainly on the specific polyphenolic fraction present in the extracts [60]. In addition, another experimental study reported the potential apoptotic and antiproliferative effects of virgin olive oil phenolic compounds and their colonic metabolites, showing that HT29 cells were more sensitive to phenol treatments than Caco-2 [61]. Based on all these observations, our work demonstrates that extracts obtained from artichoke processing waste could act as pro-apoptotic agents. These extremely inexpensive processing wastes, therefore, appear to maintain potent bioactivity, highlighting how artichoke by-products may represent a valuable source of bioactive molecules capable of directing tumor cells toward programmed cell death, making them excellent candidates for the development of nutraceuticals or adjuvants in cancer therapies. Significant biological efficacy thus emerges, since, in addition to the already documented antioxidant activity exhibited by artichoke by-products [62,63], our work demonstrates, for the first time, that extracts obtained from artichoke waste exhibit clear cytotoxic activity in colon adenocarcinoma cells, likely due to the specific combination of phenolic acids and flavonoids contained in the extracts. A broad versatility is also evident, as the extracts used in our study exert their effect on two different cell lines, suggesting that the cytotoxic action is robust and not specific to a single colon cancer subtype.

## 5. Conclusions

In conclusion, our results suggest artichoke by-products as an effective and environmentally friendly source capable of providing extracts rich in bioactive molecules and transforming waste products into resources. Artichoke by-products could represent promising sources of natural anticancer agents for potential future pharmaceutical applications, supporting the use of natural products in the treatment of cancer to protect the environment and reduce the side effects associated with conventional therapies.

Although our study highlights the anticancer potential of artichoke waste extracts, some limitations can be recognized, such as the exclusive use of in vitro cell models that cannot fully replicate the systemic complexity of a living organism. Future in vivo studies will be essential to validate the biological efficacy and bioavailability of these extracts, while also exploring the specific synergies between the various secondary metabolites for the development of targeted nutraceutical formulations or adjuvants in conventional cancer therapies.

## Figures and Tables

**Table 1:** The total phenol content and semi-quantified content, analyzed via UHPLC-DAD, of the main phenolic compounds of extracts from the four different artichoke varieties. Values for individual phenolic compounds are expressed as mg 5-*O*-caffeoylquinic acid equivalents/L.

	BriB	VaMB	LMTB	CAPB
TPC (mg GAE/L)	206.24 ± 5.35 c	269.51 ± 5.12 a	254.48 ± 2.91 b	122.70 ± 3.79 d
5-*O*-caffeoylquinic acid (mg/L)	0.63 ± 0.17 b	19.30 ± 2.77 a	1.05 ± 0.22 b	0.47 ± 0.24 b
3,4-di-*O*-caffeoylquinic acid (mg/L)	8.88 ± 0.55 b	4.87 ± 0.27 c	14.27 ± 0.38 a	9.81 ± 1.09 b
3,5-di-*O*-caffeoylquinic acid (mg/L)	0.36 ± 0.12 c	11.01 ± 0.94 a	3.91 ± 0.28 b	1.26 ± 0.46 c
Luteolin-7-*O*-rutinoside (mg/L)	0.53 ± 0.01	0.65 ± 0.22	0.29 ± 0.09	0.37 ± 0.18
Luteolin-7-*O*-glucoside (mg/L)	2.33 ± 0.45 a	0.18 ± 0.08 c	0.63 ± 0.38 bc	1.84 ± 0.85 ab
Luteolin-7-*O*-glucuronide (mg/L)	0.73 ± 0.14	0.43 ± 0.12	0.39 ± 0.03	0.75 ± 0.12
Apigenin-7-*O*-rutinoside (mg/L)	0.68 ± 0.14 b	5.48 ± 0.29 a	0.85 ± 0.07 b	0.66 ± 0.22 b
Apigenin-7-*O*-glucuronide (mg/L)	1.26 ± 0.48	0.50 ± 0.06	0.58 ± 0.14	0.44 ± 0.06
Total	15.29 ± 0.81 c	42.42 ± 1.73 a	21.96 ± 0.68 b	15.60 ± 0.47 c

All values are expressed as the mean ± SD of three replicate measurements. Different letters indicate significant differences (*p* < 0.05; one-way ANOVA).

**Table 2:** The significance between the various groups of extracts at the concentrations tested in Caco-2 cells.

Caspase 9	Caspase 3	Caspase 9	Caspase 3
2 mg/mL	2 mg/mL	3 mg/mL	3 mg/mL
*p* < 0.05 BriB vs. LMTB	*p* < 0.01 BriB vs. LMTB	*p* < 0.05 BriB vs. CAPB	*p* < 0.01 LMTB vs. CAPB
*p* < 0.01 VaMB vs. LMTB	*p* < 0.05 VaMB vs. LMTB	*p* < 0.01 VaMB vs. CAPB	*p* < 0.01 LMTB vs. CAPB
*p* < 0.01 LMTB vs. CAPB		*p* < 0.01 LMTB vs. CAPB	*p* < 0.01 LMTB vs. CAPB

**Table 3:** The significance between the various groups of extracts at the concentrations tested in HT29 cells.

Caspase 9	Caspase 3	Caspase 9	Caspase 3
2 mg/mL	2 mg/mL	3 mg/mL	3 mg/mL
*p* < 0.01 BriB vs. VaMB	*p* < 0.05 BriB vs. VaMB	*p* < 0.01 BriB vs. VaMB	*p* < 0.05 BriB vs. VaMB
*p* < 0.05 BriB vs. LMTB	*p* < 0.05 BriB vs. LMTB	*p* < 0.01 BriB vs. CAPB	*p* < 0.05 BriB vs. VaMB
*p* < 0.05 VaMB vs. CAPB	*p* < 0.05 VaMB vs. CAPB	*p* < 0.01 VaMB vs. LMTB	*p* < 0.05 BriB vs. VaMB
*p* < 0.05 LMTB vs. CAPB	*p* < 0.05 LMTB vs. CAPB		*p* < 0.01 LMTB vs. CAPB

## Data Availability

The original contributions presented in the study are included in the article. Further inquiries can be directed to the corresponding author.

## Supplementary Materials

The following supporting information can be downloaded at https://www.mdpi.com/article/10.3390/nu18132077/s1: Figure S1: Chromatographic profile (UHPLC-MS/MS) of VaMB extract and identified phenolic compounds.
