# Supplementary material for: Phenolic-Rich Extracts from Artichoke By-Products Promote Apoptosis in Human Colorectal Cancer Cell Lines
Source: Nutrients. 2026 Jun 25;18(13):2077. doi: 10.3390/nu18132077 (PMC13363651; doi:10.3390/nu18132077)
Supplement: Supplementary file 1 [file nutrients-18-02077-s001.zip › nutrients-4372498-supplementary.pdf]

Supplementary Figure S1

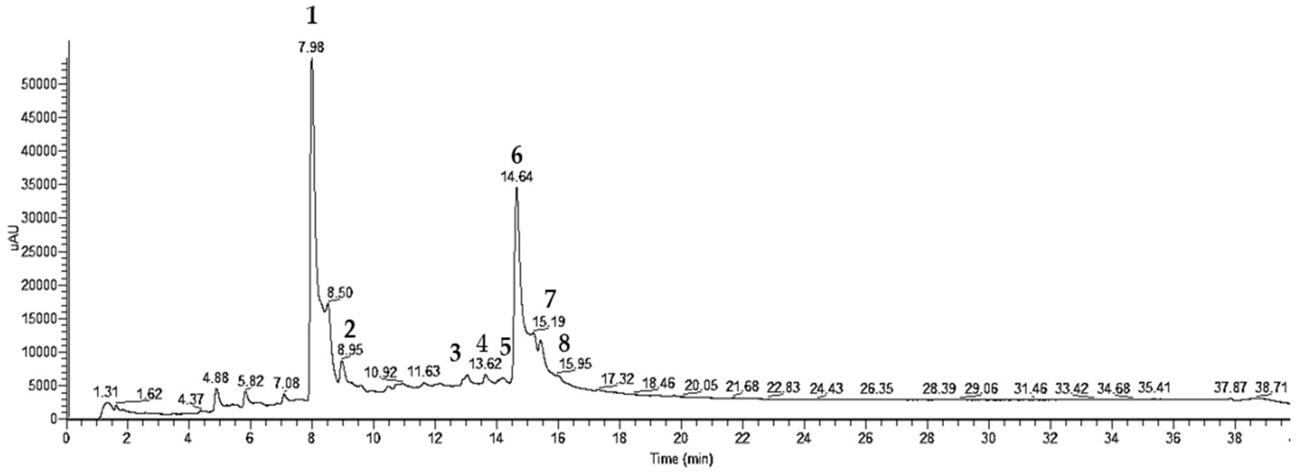

Figure S1: Chromatographic profile **(**UHPLC-MS/MS) of VaMB extract and identified phenolic compounds: (1) 5-O-caffeoylquinic acid, 353 $[M-H]^-$ (m/z) ; (2) luteolin 7-O-rutinoside, 593 $[M-H]^-$ (m/z); (3) luteolin 7-O-glucoside, 447 $[M-H]^-$ (m/z); (4) luteolin 7-O-glucuronide , 461 $[M-H]^-$ (m/z); (5) apigenin 7-O-rutinoside, 577 $[M-H]^-$ (m/z); (6) 3,5-di-O-caffeoylquinic acid, 515 $[M-H^-]$ (m/z); (7) 3,4-di-O-caffeoylquinic acid, 515 $[M-H]^-$ (m/z); (8) apigenin 7-O-glucuronide, 445 $[M-H]^-$ (m/z).
